# (*S*,*E*)-*N*-Methyl-4-[(*S*)2,6,6-trimethyl-4-oxocyclo­hex-2-en­yl]but-3-en-2-aminium chloride

**DOI:** 10.1107/S1600536811015297

**Published:** 2011-05-07

**Authors:** Liu-Shuan Chang, Hong-Quan Duan

**Affiliations:** a School of Pharmaceutical Sciences, Research Center of Basic Medical Sciences, Tianjin Medical University, Tianjin 300070, People’s Republic of China

## Abstract

The title compound, C_14_H_24_NO^+^·Cl^−^, crystallizes with four independent mol­ecules in the asymmetric unit. It was isolated from plant *Pachysandra terminalis* Siebold & Zucc. The six-membered ring has a conformation close to an envelope. In the crystal, N—H⋯Cl hydrogen-bonding inter­actions exist between secondary ammonium groups and free chloride anions, resulting in a one-dimensional supra­molecular structure oriented along [100]. The crystal studied was found to be a two-component non-merohedral twin with twin law [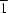
00/0
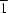
0/101], the fractional contribution of the minor component being approximately 33%.

## Related literature

For a general background to *Pachysandra terminalis* Siebold & Zucc., see: Kikuchi & Uyeo (1965 ▶, 1967*a* ▶,*b* ▶); Kikuchi *et al.* (1981 ▶); Chiu *et al.* (1990 ▶, 1992 ▶). For the determination of the twin law, see: Spek (2009 ▶).
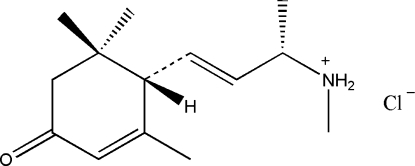


## Experimental

### 

#### Crystal data

C_14_H_24_NO^+^·Cl^−^
*M* *_r_* = 257.79
Monoclinic, 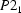

*a* = 14.547 (3) Å
*b* = 12.247 (2) Å
*c* = 19.035 (4) Å
β = 112.46 (3)°
*V* = 3134.0 (13) Å^3^
*Z* = 8
Mo *K*α radiation
μ = 0.23 mm^−1^
*T* = 113 K
0.20 × 0.12 × 0.10 mm

#### Data collection

Rigaku Saturn CCD area-detector diffractometer
Absorption correction: multi-scan (*CrystalClear*; Rigaku/MSC, 2005 ▶) *T*_min_ = 0.955, *T*_max_ = 0.977
30954 measured reflections
12371 independent reflections
10363 reflections with *I* > 2σ(*I*)
*R*_int_ = 0.046

#### Refinement

*R*[*F* ^2^ > 2σ(*F* ^2^)] = 0.047
*wR*(*F* ^2^) = 0.092
*S* = 1.01
12371 reflections
658 parameters
7 restraints
H atoms treated by a mixture of independent and constrained refinement
Δρ_max_ = 0.24 e Å^−3^
Δρ_min_ = −0.35 e Å^−3^
Absolute structure: Flack (1983 ▶), 4816 Friedel pairs
Flack parameter: 0.03 (4)

### 

Data collection: *CrystalClear* (Rigaku/MSC, 2005 ▶); cell refinement: *CrystalClear*; data reduction: *CrystalClear*; program(s) used to solve structure: *SHELXS97* (Sheldrick, 2008 ▶); program(s) used to refine structure: *SHELXL97* (Sheldrick, 2008 ▶); molecular graphics: *SHELXTL* (Sheldrick, 2008 ▶); software used to prepare material for publication: *CrystalStructure* (Rigaku/MSC, 2005 ▶) and *SHELXTL*.

## Supplementary Material

Crystal structure: contains datablocks I, global. DOI: 10.1107/S1600536811015297/bh2347sup1.cif

Structure factors: contains datablocks I. DOI: 10.1107/S1600536811015297/bh2347Isup2.hkl

Supplementary material file. DOI: 10.1107/S1600536811015297/bh2347Isup3.cml

Additional supplementary materials:  crystallographic information; 3D view; checkCIF report

## Figures and Tables

**Table 1:** Hydrogen-bond geometry (Å, °)

*D*—H⋯*A*	*D*—H	H⋯*A*	*D*⋯*A*	*D*—H⋯*A*
N1—H1*A*⋯Cl1^i^	0.93 (2)	2.19 (2)	3.109 (3)	173 (3)
N1—H1*B*⋯Cl4^i^	0.93 (2)	2.17 (2)	3.069 (4)	163 (3)
N2—H2*A*⋯Cl2	0.94 (2)	2.17 (2)	3.105 (3)	171 (3)
N2—H2*B*⋯Cl1	0.91 (2)	2.19 (2)	3.089 (4)	175 (4)
N3—H3*A*⋯Cl3^ii^	0.93 (2)	2.18 (2)	3.108 (3)	175 (3)
N3—H3*B*⋯Cl2^i^	0.92 (2)	2.18 (2)	3.088 (4)	171 (3)
N4—H4*A*⋯Cl4^iii^	0.85 (3)	2.28 (4)	3.117 (4)	169 (4)
N4—H4*B*⋯Cl3^iii^	0.96 (4)	2.13 (4)	3.083 (4)	172 (4)

Symmetry codes: (i) 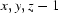; (ii) 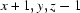; (iii) 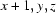.

**Fig. 1.**
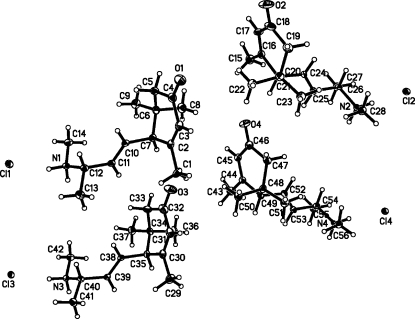
The asymmetric unit of the title compound, with 30% probability displacement ellipsoids. H atoms are shown as small spheres of arbitrary radius. There are four independent molecules in an asymmetric unit.

**Fig. 2.**
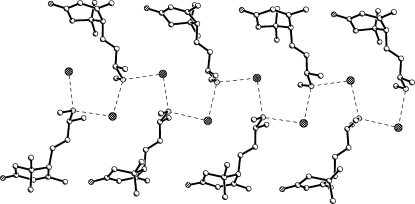
The packing of the title compound, showing the one-dimensional structure. Hydrogen bonds are drawn as dashed lines. For clarity H atoms have been omitted.

## supplementary crystallographic information

### Comment

The title substance is a new alkaloid skeleton from natural source, which is isolated from the *n*-butanol soluble fraction of *Pachysandra terminalis* Sieb. & Zucc. The plant *P. terminalis* is mainly distributed in Japan and Hubei Province of China*.* The whole plant of *P. terminalis* has been used in Chinese folk medicine to treat rheumatism, spasm of calf and abnormal menstruation. A series of alkaloids and triterpenoids have been isolated from *Pachysandra terminalis* Sieb. & Zucc. (Kikuchi & Uyeo, 1965, 1967*a*,*b*; Kikuchi *et al.*, 1981; Chiu *et al.*, 1990, 1992). In order to find its bioactive ingredients we studied the chemical constituents and found the title compound.

Four independent molecules can be observed in an asymmetric unit (Fig. 1). The NH_2_^+^ groups in the molecules act as an hydrogen-bond donors to Cl^-^ ions, forming N—H···Cl hydrogen bonds, resulting in a one-dimensional supramolecular structure (Fig. 2).

### Experimental

Plant material: *Pachysandra terminalis* Sieb. & Zucc. was collected from the Hefeng area in Hubei Province of China and authenticated by Professor Ding-Rong Wan (School of Life Sciences, South-Central University For Nationalities). Extraction and Isolation: The dry powders (8 kg) of the whole plant of *P. terminalis* were refluxed for 6 h with 95% EtOH three times. After removal of the EtOH under reduced pressure, the extract was suspended in water and then partitioned with petroleum ether, EtOAc and *n*-butanol. The *n*-butanol soluble fraction (360 g) was subjected to macroporous resin column chromatography with a gradient of EtOH-H_2_O (1:9 → 19:1 *v/v*) to afford five fractions (F_1_ to F_5_), pooled by common TLC characteristics. F_2_ (9.6 g) was separated by a Toyopeal HW-40 column (CH_2_Cl_2_—MeOH, 1:1 *v/v*) to afford four fractions (F_2.1_ to F_2.4_). F_2.2_ was separated by preparative HPLC with MeOH to give three fractions (F_2.2.1_ to F_2.2.3_). F_2.2.1_ was separated by preparative HPLC (MeOH-H_2_O, 3:7 *v/v*) to afford the title compound. Colourless block crystals were obtained by slow evaporation from a methanol solution of the product, at room temperature.

### Refinement

Secondary ammonium H atoms were located in a difference Fourier map and refined using restrained N—H bond lengths for N1, N2, and N3 [target value: 0.90 (2) Å]. Other H atoms were positioned geometrically and refined using a riding model, with C—H = 0.95–0.99 Å and *U*_iso_(H) = 1.5*U*_eq_(carrier C) for methyl and 1.2*U*_eq_(carrier C) for the others. For the treatment of twinned diffraction data, see: Spek (2009).

### Crystal data

**Table:**

C_14_H_24_NO^+^·Cl^−^	*F*(000) = 1120
*M**_r_* = 257.79	*D*_x_ = 1.093 Mg m^−^^3^
Monoclinic, *P*2_1_	Mo *K*α radiation, λ = 0.71073 Å
Hall symbol: P 2yb	Cell parameters from 9688 reflections
*a* = 14.547 (3) Å	θ = 2.9–27.5°
*b* = 12.247 (2) Å	µ = 0.23 mm^−^^1^
*c* = 19.035 (4) Å	*T* = 113 K
β = 112.46 (3)°	Block, colourless
*V* = 3134.0 (13) Å^3^	0.20 × 0.12 × 0.10 mm
*Z* = 8	

### Data collection

**Table:**

Rigaku Saturn CCD area-detector diffractometer	12371 independent reflections
Radiation source: rotating anode	10363 reflections with *I* > 2σ(*I*)
multilayer	*R*_int_ = 0.046
Detector resolution: 7.31 pixels mm^-1^	θ_max_ = 27.5°, θ_min_ = 1.7°
ω and φ scans	*h* = −18→18
Absorption correction: multi-scan (*CrystalClear*; Rigaku/MSC, 2005)	*k* = −15→14
*T*_min_ = 0.955, *T*_max_ = 0.977	*l* = −24→24
30954 measured reflections	

### Refinement

**Table:**

Refinement on *F*^2^	Secondary atom site location: difference Fourier map
Least-squares matrix: full	Hydrogen site location: inferred from neighbouring sites
*R*[*F*^2^ > 2σ(*F*^2^)] = 0.047	H atoms treated by a mixture of independent and constrained refinement
*wR*(*F*^2^) = 0.092	*w* = 1/[σ^2^(*F*_o_^2^) + (0.0421*P*)^2^] where *P* = (*F*_o_^2^ + 2*F*_c_^2^)/3
*S* = 1.01	(Δ/σ)_max_ = 0.001
12371 reflections	Δρ_max_ = 0.24 e Å^−^^3^
658 parameters	Δρ_min_ = −0.34 e Å^−^^3^
7 restraints	Absolute structure: Flack (1983), 4816 Friedel pairs
0 constraints	Flack parameter: 0.03 (4)
Primary atom site location: structure-invariant direct methods	

### Fractional atomic coordinates and isotropic or equivalent isotropic displacement parameters (Å^2^)

**Table:**

	*x*	*y*	*z*	*U*_iso_*/*U*_eq_	
Cl1	0.60208 (7)	0.40656 (9)	0.85070 (5)	0.0284 (3)	
Cl2	0.75173 (7)	0.36146 (9)	0.65009 (5)	0.0254 (3)	
Cl3	0.10038 (7)	0.36764 (9)	0.84472 (5)	0.0276 (3)	
Cl4	0.25369 (7)	0.31672 (9)	0.65341 (6)	0.0290 (3)	
N1	0.3768 (2)	0.3827 (3)	−0.18164 (19)	0.0237 (8)	
H1A	0.4425 (16)	0.392 (3)	−0.176 (2)	0.028*	
H1B	0.352 (2)	0.355 (3)	−0.2308 (13)	0.028*	
N2	0.5596 (3)	0.3447 (3)	0.68389 (19)	0.0226 (8)	
H2A	0.615 (2)	0.356 (3)	0.671 (2)	0.027*	
H2B	0.572 (3)	0.359 (3)	0.7333 (12)	0.027*	
N3	0.8777 (2)	0.3890 (3)	−0.17864 (18)	0.0219 (8)	
H3A	0.9452 (15)	0.386 (3)	−0.169 (2)	0.026*	
H3B	0.847 (2)	0.378 (3)	−0.2302 (11)	0.026*	
N4	1.0515 (3)	0.3390 (3)	0.6733 (2)	0.0237 (8)	
H4A	1.102 (3)	0.329 (3)	0.662 (2)	0.028*	
H4B	1.064 (3)	0.355 (3)	0.726 (2)	0.028*	
O1	0.1485 (2)	0.2664 (3)	0.13065 (18)	0.0516 (9)	
O2	0.0107 (2)	0.3327 (3)	0.3666 (2)	0.0679 (12)	
O3	0.6463 (3)	0.3929 (3)	0.1392 (2)	0.0620 (11)	
O4	0.5144 (2)	0.4635 (3)	0.36417 (18)	0.0535 (9)	
C1	0.4766 (3)	0.1922 (3)	0.1329 (2)	0.0366 (10)	
H1C	0.4704	0.1260	0.1598	0.055*	
H1D	0.5352	0.2336	0.1650	0.055*	
H1E	0.4837	0.1718	0.0855	0.055*	
C2	0.3856 (3)	0.2610 (3)	0.1153 (2)	0.0264 (9)	
C3	0.3091 (3)	0.2310 (4)	0.1332 (2)	0.0330 (10)	
H3	0.3130	0.1635	0.1588	0.040*	
C4	0.2191 (3)	0.2975 (4)	0.1151 (2)	0.0332 (10)	
C5	0.2141 (3)	0.4036 (3)	0.0735 (2)	0.0288 (9)	
H5A	0.1752	0.4568	0.0899	0.035*	
H5B	0.1778	0.3908	0.0184	0.035*	
C6	0.3160 (3)	0.4543 (3)	0.0858 (2)	0.0269 (9)	
C7	0.3836 (3)	0.3657 (4)	0.0718 (2)	0.0240 (8)	
H7	0.4529	0.3950	0.0889	0.029*	
C8	0.3653 (3)	0.4953 (4)	0.1683 (2)	0.0388 (11)	
H8A	0.3754	0.4336	0.2033	0.058*	
H8B	0.3222	0.5496	0.1782	0.058*	
H8C	0.4297	0.5286	0.1760	0.058*	
C9	0.3024 (3)	0.5500 (3)	0.0317 (2)	0.0374 (9)	
H9A	0.2632	0.6071	0.0434	0.056*	
H9B	0.2675	0.5251	−0.0207	0.056*	
H9C	0.3676	0.5793	0.0377	0.056*	
C10	0.3498 (3)	0.3350 (3)	−0.0105 (2)	0.0226 (9)	
H10	0.2809	0.3207	−0.0366	0.027*	
C11	0.4069 (3)	0.3259 (3)	−0.0505 (2)	0.0232 (9)	
H11	0.4756	0.3429	−0.0264	0.028*	
C12	0.3682 (3)	0.2903 (3)	−0.1310 (2)	0.0241 (9)	
H12	0.2963	0.2715	−0.1465	0.029*	
C13	0.4215 (3)	0.1921 (4)	−0.1461 (2)	0.0346 (10)	
H13A	0.4920	0.2095	−0.1320	0.052*	
H13B	0.3918	0.1734	−0.2002	0.052*	
H13C	0.4150	0.1300	−0.1159	0.052*	
C14	0.3322 (3)	0.4880 (4)	−0.1721 (3)	0.0301 (10)	
H14A	0.3730	0.5202	−0.1229	0.045*	
H14B	0.2648	0.4751	−0.1740	0.045*	
H14C	0.3294	0.5381	−0.2130	0.045*	
C15	0.2916 (3)	0.5499 (3)	0.3735 (2)	0.0402 (11)	
H15A	0.3446	0.5748	0.4205	0.060*	
H15B	0.2413	0.6074	0.3541	0.060*	
H15C	0.3199	0.5335	0.3355	0.060*	
C16	0.2445 (3)	0.4494 (3)	0.3893 (2)	0.0286 (9)	
C17	0.1473 (3)	0.4395 (4)	0.3716 (2)	0.0368 (11)	
H17	0.1057	0.4996	0.3480	0.044*	
C18	0.1012 (3)	0.3409 (4)	0.3864 (3)	0.0432 (13)	
C19	0.1689 (3)	0.2496 (3)	0.4276 (2)	0.0364 (10)	
H19A	0.1330	0.1795	0.4117	0.044*	
H19B	0.1856	0.2581	0.4828	0.044*	
C20	0.2662 (3)	0.2444 (3)	0.4133 (2)	0.0289 (9)	
C21	0.3162 (3)	0.3575 (4)	0.4300 (2)	0.0216 (8)	
H21	0.3718	0.3574	0.4113	0.026*	
C22	0.2423 (3)	0.2147 (4)	0.3300 (2)	0.0356 (10)	
H22A	0.1941	0.2669	0.2969	0.053*	
H22B	0.2142	0.1409	0.3200	0.053*	
H22C	0.3035	0.2172	0.3198	0.053*	
C23	0.3339 (3)	0.1579 (3)	0.4649 (2)	0.0340 (9)	
H23A	0.3059	0.0853	0.4479	0.051*	
H23B	0.3393	0.1699	0.5173	0.051*	
H23C	0.4000	0.1629	0.4628	0.051*	
C24	0.3593 (3)	0.3841 (3)	0.5135 (2)	0.0207 (8)	
H24	0.3143	0.3850	0.5388	0.025*	
C25	0.4529 (3)	0.4062 (3)	0.5550 (2)	0.0238 (9)	
H25	0.4998	0.4066	0.5313	0.029*	
C26	0.4881 (3)	0.4308 (3)	0.6381 (2)	0.0198 (8)	
H26	0.4291	0.4307	0.6529	0.024*	
C27	0.5398 (3)	0.5411 (3)	0.6596 (2)	0.0288 (9)	
H27A	0.4941	0.5991	0.6317	0.043*	
H27B	0.5989	0.5421	0.6467	0.043*	
H27C	0.5598	0.5530	0.7144	0.043*	
C28	0.5207 (3)	0.2325 (3)	0.6718 (3)	0.0302 (10)	
H28A	0.5089	0.2095	0.6198	0.045*	
H28B	0.4580	0.2297	0.6798	0.045*	
H28C	0.5691	0.1833	0.7079	0.045*	
C29	0.9222 (3)	0.1765 (4)	0.1326 (3)	0.0425 (11)	
H29A	0.8918	0.1190	0.1523	0.064*	
H29B	0.9880	0.1945	0.1707	0.064*	
H29C	0.9290	0.1509	0.0860	0.064*	
C30	0.8585 (3)	0.2750 (3)	0.1154 (2)	0.0281 (9)	
C31	0.7785 (3)	0.2854 (4)	0.1344 (2)	0.0365 (10)	
H31	0.7606	0.2254	0.1582	0.044*	
C32	0.7182 (3)	0.3840 (4)	0.1200 (3)	0.0409 (12)	
C33	0.7455 (3)	0.4763 (3)	0.0787 (2)	0.0352 (10)	
H33A	0.7268	0.5464	0.0956	0.042*	
H33B	0.7059	0.4689	0.0236	0.042*	
C34	0.8556 (3)	0.4805 (3)	0.0915 (2)	0.0279 (9)	
C35	0.8889 (3)	0.3661 (4)	0.0745 (2)	0.0228 (8)	
H35	0.9631	0.3660	0.0925	0.027*	
C36	0.9168 (3)	0.5104 (4)	0.1747 (2)	0.0381 (11)	
H36A	0.9871	0.5171	0.1824	0.057*	
H36B	0.9092	0.4532	0.2081	0.057*	
H36C	0.8931	0.5801	0.1868	0.057*	
C37	0.8720 (3)	0.5684 (3)	0.0399 (2)	0.0345 (9)	
H37A	0.9404	0.5639	0.0423	0.052*	
H37B	0.8605	0.6407	0.0570	0.052*	
H37C	0.8255	0.5566	−0.0126	0.052*	
C38	0.8472 (3)	0.3421 (3)	−0.0090 (2)	0.0219 (9)	
H38	0.7768	0.3428	−0.0339	0.026*	
C39	0.8991 (3)	0.3197 (3)	−0.0518 (2)	0.0205 (8)	
H39	0.9696	0.3167	−0.0282	0.025*	
C40	0.8508 (3)	0.2994 (4)	−0.1352 (2)	0.0231 (9)	
H40	0.7771	0.3003	−0.1498	0.028*	
C41	0.8787 (3)	0.1929 (3)	−0.1608 (2)	0.0316 (10)	
H41A	0.8606	0.1326	−0.1348	0.047*	
H41B	0.9506	0.1915	−0.1484	0.047*	
H41C	0.8432	0.1852	−0.2158	0.047*	
C42	0.8539 (3)	0.5010 (3)	−0.1619 (3)	0.0293 (10)	
H42A	0.8986	0.5216	−0.1104	0.044*	
H42B	0.7849	0.5037	−0.1656	0.044*	
H42C	0.8624	0.5520	−0.1987	0.044*	
C43	0.8362 (3)	0.5329 (4)	0.3550 (3)	0.0460 (12)	
H43A	0.8966	0.5460	0.4002	0.069*	
H43B	0.8053	0.6030	0.3340	0.069*	
H43C	0.8534	0.4937	0.3168	0.069*	
C44	0.7644 (3)	0.4655 (3)	0.3766 (2)	0.0273 (9)	
C45	0.6707 (3)	0.4961 (4)	0.3592 (2)	0.0325 (10)	
H45	0.6487	0.5624	0.3321	0.039*	
C46	0.5998 (3)	0.4324 (4)	0.3800 (2)	0.0334 (10)	
C47	0.6372 (3)	0.3269 (3)	0.4227 (2)	0.0300 (10)	
H47A	0.5819	0.2735	0.4071	0.036*	
H47B	0.6566	0.3409	0.4777	0.036*	
C48	0.7266 (3)	0.2755 (3)	0.4099 (2)	0.0262 (9)	
C49	0.8074 (3)	0.3643 (4)	0.4227 (2)	0.0228 (8)	
H49	0.8594	0.3341	0.4057	0.027*	
C50	0.6942 (3)	0.2327 (4)	0.3283 (2)	0.0372 (10)	
H50A	0.6652	0.2925	0.2925	0.056*	
H50B	0.6447	0.1748	0.3200	0.056*	
H50C	0.7522	0.2034	0.3203	0.056*	
C51	0.7669 (3)	0.1809 (3)	0.4647 (2)	0.0343 (9)	
H51A	0.7173	0.1224	0.4520	0.051*	
H51B	0.7816	0.2060	0.5168	0.051*	
H51C	0.8280	0.1534	0.4607	0.051*	
C52	0.8574 (3)	0.3972 (3)	0.5053 (2)	0.0238 (9)	
H52	0.8153	0.4150	0.5314	0.029*	
C53	0.9535 (3)	0.4034 (3)	0.5440 (2)	0.0249 (9)	
H53	0.9977	0.3878	0.5193	0.030*	
C54	0.9954 (3)	0.4344 (4)	0.6264 (2)	0.0253 (9)	
H54	0.9389	0.4526	0.6421	0.030*	
C55	1.0666 (3)	0.5323 (3)	0.6449 (3)	0.0337 (10)	
H55A	1.0318	0.5958	0.6152	0.051*	
H55B	1.1239	0.5145	0.6320	0.051*	
H55C	1.0894	0.5492	0.6992	0.051*	
C56	0.9936 (3)	0.2359 (4)	0.6599 (3)	0.0304 (10)	
H56A	0.9828	0.2078	0.6092	0.046*	
H56B	0.9292	0.2500	0.6635	0.046*	
H56C	1.0305	0.1818	0.6983	0.046*	

### Atomic displacement parameters (Å^2^)

**Table:**

	*U*^11^	*U*^22^	*U*^33^	*U*^12^	*U*^13^	*U*^23^
Cl1	0.0179 (5)	0.0417 (8)	0.0252 (5)	0.0000 (4)	0.0078 (4)	−0.0020 (4)
Cl2	0.0206 (5)	0.0351 (7)	0.0226 (5)	0.0003 (4)	0.0106 (4)	0.0000 (4)
Cl3	0.0185 (5)	0.0392 (7)	0.0248 (5)	0.0008 (4)	0.0080 (4)	−0.0003 (4)
Cl4	0.0230 (6)	0.0406 (7)	0.0269 (5)	−0.0018 (4)	0.0134 (4)	−0.0010 (4)
N1	0.0176 (18)	0.030 (2)	0.0265 (18)	0.0024 (15)	0.0120 (15)	0.0018 (15)
N2	0.0191 (18)	0.029 (2)	0.0218 (17)	−0.0008 (14)	0.0102 (15)	0.0012 (14)
N3	0.0191 (18)	0.028 (2)	0.0191 (16)	0.0032 (14)	0.0081 (14)	0.0016 (13)
N4	0.0168 (18)	0.033 (2)	0.0254 (18)	0.0012 (14)	0.0129 (15)	−0.0003 (14)
O1	0.0412 (19)	0.064 (2)	0.059 (2)	−0.0070 (17)	0.0292 (18)	0.0095 (16)
O2	0.0257 (19)	0.088 (3)	0.088 (3)	0.0065 (18)	0.0204 (19)	−0.017 (2)
O3	0.041 (2)	0.078 (3)	0.083 (3)	−0.0012 (18)	0.041 (2)	−0.009 (2)
O4	0.0293 (18)	0.066 (2)	0.062 (2)	0.0187 (16)	0.0130 (16)	0.0087 (17)
C1	0.031 (2)	0.034 (2)	0.039 (2)	0.0032 (19)	0.0058 (19)	0.0162 (18)
C2	0.028 (2)	0.028 (2)	0.0179 (18)	−0.0049 (17)	0.0033 (17)	−0.0008 (16)
C3	0.038 (3)	0.030 (2)	0.028 (2)	−0.0030 (19)	0.0090 (19)	0.0056 (17)
C4	0.033 (2)	0.039 (3)	0.029 (2)	−0.007 (2)	0.0120 (19)	−0.0046 (19)
C5	0.026 (2)	0.031 (2)	0.029 (2)	0.0001 (16)	0.0099 (17)	−0.0053 (18)
C6	0.029 (2)	0.027 (2)	0.0215 (18)	−0.0040 (17)	0.0063 (18)	−0.0056 (15)
C7	0.020 (2)	0.026 (2)	0.0244 (19)	−0.0009 (18)	0.0070 (17)	0.0034 (18)
C8	0.038 (3)	0.042 (3)	0.033 (2)	−0.009 (2)	0.010 (2)	−0.011 (2)
C9	0.044 (3)	0.026 (2)	0.039 (2)	0.0007 (18)	0.013 (2)	0.0015 (17)
C10	0.016 (2)	0.024 (2)	0.027 (2)	0.0012 (15)	0.0068 (17)	0.0030 (15)
C11	0.016 (2)	0.027 (2)	0.027 (2)	0.0002 (16)	0.0085 (17)	0.0008 (16)
C12	0.021 (2)	0.027 (2)	0.028 (2)	−0.0018 (17)	0.0133 (18)	0.0032 (16)
C13	0.037 (3)	0.033 (2)	0.038 (2)	0.000 (2)	0.019 (2)	−0.0003 (18)
C14	0.027 (2)	0.029 (2)	0.037 (2)	0.0043 (18)	0.014 (2)	0.0056 (18)
C15	0.050 (3)	0.035 (2)	0.038 (2)	0.010 (2)	0.019 (2)	0.007 (2)
C16	0.036 (3)	0.027 (2)	0.0227 (19)	0.0071 (19)	0.0119 (18)	0.0013 (16)
C17	0.027 (3)	0.044 (3)	0.036 (2)	0.016 (2)	0.0094 (19)	−0.006 (2)
C18	0.026 (3)	0.060 (4)	0.048 (3)	−0.005 (2)	0.019 (2)	−0.017 (2)
C19	0.031 (2)	0.043 (3)	0.035 (2)	−0.0109 (19)	0.0123 (19)	−0.0105 (19)
C20	0.033 (2)	0.023 (2)	0.030 (2)	−0.0011 (17)	0.0111 (18)	−0.0050 (16)
C21	0.022 (2)	0.026 (2)	0.0203 (18)	0.0018 (17)	0.0113 (17)	0.0001 (16)
C22	0.042 (3)	0.032 (2)	0.026 (2)	0.0066 (19)	0.0060 (19)	−0.0070 (19)
C23	0.042 (2)	0.027 (2)	0.032 (2)	0.0025 (18)	0.012 (2)	−0.0025 (16)
C24	0.023 (2)	0.024 (2)	0.0158 (17)	0.0034 (16)	0.0090 (16)	0.0026 (14)
C25	0.028 (2)	0.024 (2)	0.024 (2)	0.0002 (17)	0.0146 (18)	0.0030 (17)
C26	0.014 (2)	0.023 (2)	0.0238 (19)	0.0008 (15)	0.0090 (16)	−0.0020 (16)
C27	0.031 (2)	0.023 (2)	0.030 (2)	−0.0017 (17)	0.0080 (18)	−0.0024 (16)
C28	0.032 (2)	0.024 (2)	0.034 (2)	0.0020 (18)	0.0115 (19)	0.0044 (18)
C29	0.047 (3)	0.034 (3)	0.045 (3)	0.006 (2)	0.016 (2)	0.014 (2)
C30	0.030 (2)	0.030 (2)	0.0215 (18)	−0.0082 (18)	0.0063 (18)	−0.0021 (16)
C31	0.043 (3)	0.038 (3)	0.032 (2)	−0.008 (2)	0.017 (2)	−0.0004 (19)
C32	0.032 (2)	0.054 (3)	0.040 (2)	−0.014 (2)	0.018 (2)	−0.012 (2)
C33	0.031 (2)	0.036 (2)	0.040 (2)	0.0076 (19)	0.015 (2)	−0.0050 (19)
C34	0.032 (2)	0.029 (2)	0.0228 (19)	−0.0003 (17)	0.0109 (18)	−0.0067 (16)
C35	0.022 (2)	0.026 (2)	0.0197 (18)	−0.0029 (18)	0.0071 (16)	0.0013 (17)
C36	0.048 (3)	0.043 (3)	0.023 (2)	−0.015 (2)	0.013 (2)	−0.010 (2)
C37	0.051 (3)	0.023 (2)	0.031 (2)	0.0019 (18)	0.017 (2)	−0.0019 (16)
C38	0.015 (2)	0.024 (2)	0.026 (2)	0.0024 (15)	0.0057 (17)	0.0000 (15)
C39	0.015 (2)	0.022 (2)	0.0227 (19)	0.0016 (16)	0.0058 (16)	0.0011 (16)
C40	0.023 (2)	0.027 (2)	0.0220 (18)	0.0021 (18)	0.0111 (17)	−0.0011 (16)
C41	0.040 (3)	0.028 (2)	0.031 (2)	0.002 (2)	0.018 (2)	−0.0007 (17)
C42	0.029 (2)	0.025 (2)	0.035 (2)	0.0038 (18)	0.014 (2)	−0.0007 (17)
C43	0.051 (3)	0.043 (3)	0.056 (3)	0.011 (2)	0.034 (3)	0.019 (2)
C44	0.031 (2)	0.028 (2)	0.025 (2)	0.0071 (17)	0.0129 (18)	0.0021 (16)
C45	0.035 (2)	0.036 (2)	0.026 (2)	0.0108 (19)	0.0118 (18)	0.0046 (17)
C46	0.031 (2)	0.037 (3)	0.028 (2)	−0.001 (2)	0.0060 (18)	−0.0065 (18)
C47	0.027 (2)	0.035 (3)	0.030 (2)	−0.0069 (17)	0.0123 (18)	−0.0031 (18)
C48	0.032 (2)	0.022 (2)	0.0230 (18)	0.0019 (16)	0.0084 (17)	0.0026 (15)
C49	0.023 (2)	0.027 (2)	0.0200 (18)	−0.0007 (18)	0.0101 (16)	−0.0015 (17)
C50	0.043 (3)	0.039 (3)	0.024 (2)	0.001 (2)	0.007 (2)	−0.0095 (18)
C51	0.042 (2)	0.025 (2)	0.035 (2)	0.0007 (18)	0.014 (2)	0.0021 (17)
C52	0.029 (2)	0.023 (2)	0.0232 (19)	0.0031 (17)	0.0140 (18)	0.0017 (15)
C53	0.026 (2)	0.029 (2)	0.0242 (19)	0.0005 (17)	0.0143 (18)	0.0026 (16)
C54	0.018 (2)	0.032 (2)	0.027 (2)	0.0056 (17)	0.0090 (17)	0.0042 (17)
C55	0.027 (2)	0.026 (2)	0.044 (2)	−0.0027 (18)	0.009 (2)	0.0006 (18)
C56	0.027 (2)	0.032 (2)	0.033 (2)	0.0019 (18)	0.0116 (19)	0.0018 (18)

### Geometric parameters (Å, °)

**Table:**

N1—C14	1.486 (5)	C25—C26	1.495 (5)
N1—C12	1.522 (5)	C25—H25	0.9500
N1—H1A	0.928 (18)	C26—C27	1.524 (6)
N1—H1B	0.927 (18)	C26—H26	1.0000
N2—C28	1.471 (5)	C27—H27A	0.9800
N2—C26	1.504 (5)	C27—H27B	0.9800
N2—H2A	0.940 (18)	C27—H27C	0.9800
N2—H2B	0.905 (18)	C28—H28A	0.9800
N3—C42	1.479 (5)	C28—H28B	0.9800
N3—C40	1.512 (5)	C28—H28C	0.9800
N3—H3A	0.929 (18)	C29—C30	1.480 (6)
N3—H3B	0.921 (18)	C29—H29A	0.9800
N4—C56	1.485 (5)	C29—H29B	0.9800
N4—C54	1.507 (5)	C29—H29C	0.9800
N4—H4A	0.85 (3)	C30—C31	1.349 (6)
N4—H4B	0.96 (4)	C30—C35	1.520 (5)
O1—C4	1.234 (4)	C31—C32	1.456 (7)
O2—C18	1.228 (5)	C31—H31	0.9500
O3—C32	1.237 (5)	C32—C33	1.513 (6)
O4—C46	1.222 (5)	C33—C34	1.527 (6)
C1—C2	1.496 (6)	C33—H33A	0.9900
C1—H1C	0.9800	C33—H33B	0.9900
C1—H1D	0.9800	C34—C37	1.536 (5)
C1—H1E	0.9800	C34—C36	1.537 (5)
C2—C3	1.333 (5)	C34—C35	1.556 (6)
C2—C7	1.521 (6)	C35—C38	1.497 (5)
C3—C4	1.467 (6)	C35—H35	1.0000
C3—H3	0.9500	C36—H36A	0.9800
C4—C5	1.508 (6)	C36—H36B	0.9800
C5—C6	1.540 (5)	C36—H36C	0.9800
C5—H5A	0.9900	C37—H37A	0.9800
C5—H5B	0.9900	C37—H37B	0.9800
C6—C9	1.522 (5)	C37—H37C	0.9800
C6—C8	1.539 (5)	C38—C39	1.335 (5)
C6—C7	1.555 (6)	C38—H38	0.9500
C7—C10	1.499 (5)	C39—C40	1.491 (5)
C7—H7	1.0000	C39—H39	0.9500
C8—H8A	0.9800	C40—C41	1.501 (6)
C8—H8B	0.9800	C40—H40	1.0000
C8—H8C	0.9800	C41—H41A	0.9800
C9—H9A	0.9800	C41—H41B	0.9800
C9—H9B	0.9800	C41—H41C	0.9800
C9—H9C	0.9800	C42—H42A	0.9800
C10—C11	1.328 (5)	C42—H42B	0.9800
C10—H10	0.9500	C42—H42C	0.9800
C11—C12	1.482 (6)	C43—C44	1.506 (6)
C11—H11	0.9500	C43—H43A	0.9800
C12—C13	1.516 (6)	C43—H43B	0.9800
C12—H12	1.0000	C43—H43C	0.9800
C13—H13A	0.9800	C44—C45	1.328 (5)
C13—H13B	0.9800	C44—C49	1.509 (6)
C13—H13C	0.9800	C45—C46	1.464 (6)
C14—H14A	0.9800	C45—H45	0.9500
C14—H14B	0.9800	C46—C47	1.512 (6)
C14—H14C	0.9800	C47—C48	1.545 (5)
C15—C16	1.495 (6)	C47—H47A	0.9900
C15—H15A	0.9800	C47—H47B	0.9900
C15—H15B	0.9800	C48—C51	1.519 (5)
C15—H15C	0.9800	C48—C50	1.533 (5)
C16—C17	1.327 (6)	C48—C49	1.551 (6)
C16—C21	1.529 (6)	C49—C52	1.512 (5)
C17—C18	1.460 (7)	C49—H49	1.0000
C17—H17	0.9500	C50—H50A	0.9800
C18—C19	1.499 (6)	C50—H50B	0.9800
C19—C20	1.542 (5)	C50—H50C	0.9800
C19—H19A	0.9900	C51—H51A	0.9800
C19—H19B	0.9900	C51—H51B	0.9800
C20—C23	1.523 (5)	C51—H51C	0.9800
C20—C22	1.532 (5)	C52—C53	1.309 (6)
C20—C21	1.540 (6)	C52—H52	0.9500
C21—C24	1.506 (5)	C53—C54	1.499 (6)
C21—H21	1.0000	C53—H53	0.9500
C22—H22A	0.9800	C54—C55	1.535 (6)
C22—H22B	0.9800	C54—H54	1.0000
C22—H22C	0.9800	C55—H55A	0.9800
C23—H23A	0.9800	C55—H55B	0.9800
C23—H23B	0.9800	C55—H55C	0.9800
C23—H23C	0.9800	C56—H56A	0.9800
C24—C25	1.315 (6)	C56—H56B	0.9800
C24—H24	0.9500	C56—H56C	0.9800
			
C14—N1—C12	115.0 (3)	C26—C27—H27A	109.5
C14—N1—H1A	111 (2)	C26—C27—H27B	109.5
C12—N1—H1A	110 (2)	H27A—C27—H27B	109.5
C14—N1—H1B	115 (2)	C26—C27—H27C	109.5
C12—N1—H1B	106 (2)	H27A—C27—H27C	109.5
H1A—N1—H1B	99 (3)	H27B—C27—H27C	109.5
C28—N2—C26	115.1 (3)	N2—C28—H28A	109.5
C28—N2—H2A	115 (2)	N2—C28—H28B	109.5
C26—N2—H2A	102 (2)	H28A—C28—H28B	109.5
C28—N2—H2B	105 (3)	N2—C28—H28C	109.5
C26—N2—H2B	107 (3)	H28A—C28—H28C	109.5
H2A—N2—H2B	114 (3)	H28B—C28—H28C	109.5
C42—N3—C40	115.1 (3)	C30—C29—H29A	109.5
C42—N3—H3A	108 (2)	C30—C29—H29B	109.5
C40—N3—H3A	110 (2)	H29A—C29—H29B	109.5
C42—N3—H3B	108 (2)	C30—C29—H29C	109.5
C40—N3—H3B	111 (2)	H29A—C29—H29C	109.5
H3A—N3—H3B	105 (3)	H29B—C29—H29C	109.5
C56—N4—C54	114.5 (3)	C31—C30—C29	123.4 (4)
C56—N4—H4A	109 (3)	C31—C30—C35	121.2 (4)
C54—N4—H4A	107 (3)	C29—C30—C35	115.5 (3)
C56—N4—H4B	103 (2)	C30—C31—C32	123.2 (4)
C54—N4—H4B	108 (2)	C30—C31—H31	118.4
H4A—N4—H4B	117 (3)	C32—C31—H31	118.4
C2—C1—H1C	109.5	O3—C32—C31	122.3 (4)
C2—C1—H1D	109.5	O3—C32—C33	120.5 (4)
H1C—C1—H1D	109.5	C31—C32—C33	117.3 (3)
C2—C1—H1E	109.5	C32—C33—C34	114.2 (3)
H1C—C1—H1E	109.5	C32—C33—H33A	108.7
H1D—C1—H1E	109.5	C34—C33—H33A	108.7
C3—C2—C1	122.8 (4)	C32—C33—H33B	108.7
C3—C2—C7	121.9 (4)	C34—C33—H33B	108.7
C1—C2—C7	115.3 (3)	H33A—C33—H33B	107.6
C2—C3—C4	122.9 (4)	C33—C34—C37	109.4 (3)
C2—C3—H3	118.5	C33—C34—C36	109.3 (3)
C4—C3—H3	118.5	C37—C34—C36	108.8 (3)
O1—C4—C3	121.5 (4)	C33—C34—C35	109.1 (3)
O1—C4—C5	121.0 (4)	C37—C34—C35	111.2 (3)
C3—C4—C5	117.5 (3)	C36—C34—C35	109.0 (3)
C4—C5—C6	114.7 (3)	C38—C35—C30	108.7 (3)
C4—C5—H5A	108.6	C38—C35—C34	111.4 (3)
C6—C5—H5A	108.6	C30—C35—C34	112.3 (3)
C4—C5—H5B	108.6	C38—C35—H35	108.1
C6—C5—H5B	108.6	C30—C35—H35	108.1
H5A—C5—H5B	107.6	C34—C35—H35	108.1
C9—C6—C8	109.2 (3)	C34—C36—H36A	109.5
C9—C6—C5	109.8 (3)	C34—C36—H36B	109.5
C8—C6—C5	109.5 (3)	H36A—C36—H36B	109.5
C9—C6—C7	110.4 (3)	C34—C36—H36C	109.5
C8—C6—C7	108.9 (3)	H36A—C36—H36C	109.5
C5—C6—C7	109.0 (3)	H36B—C36—H36C	109.5
C10—C7—C2	106.8 (3)	C34—C37—H37A	109.5
C10—C7—C6	112.2 (3)	C34—C37—H37B	109.5
C2—C7—C6	112.1 (3)	H37A—C37—H37B	109.5
C10—C7—H7	108.5	C34—C37—H37C	109.5
C2—C7—H7	108.5	H37A—C37—H37C	109.5
C6—C7—H7	108.5	H37B—C37—H37C	109.5
C6—C8—H8A	109.5	C39—C38—C35	126.4 (4)
C6—C8—H8B	109.5	C39—C38—H38	116.8
H8A—C8—H8B	109.5	C35—C38—H38	116.8
C6—C8—H8C	109.5	C38—C39—C40	122.5 (4)
H8A—C8—H8C	109.5	C38—C39—H39	118.7
H8B—C8—H8C	109.5	C40—C39—H39	118.7
C6—C9—H9A	109.5	C39—C40—C41	114.5 (4)
C6—C9—H9B	109.5	C39—C40—N3	110.2 (3)
H9A—C9—H9B	109.5	C41—C40—N3	107.1 (3)
C6—C9—H9C	109.5	C39—C40—H40	108.3
H9A—C9—H9C	109.5	C41—C40—H40	108.3
H9B—C9—H9C	109.5	N3—C40—H40	108.3
C11—C10—C7	126.3 (4)	C40—C41—H41A	109.5
C11—C10—H10	116.8	C40—C41—H41B	109.5
C7—C10—H10	116.8	H41A—C41—H41B	109.5
C10—C11—C12	123.0 (4)	C40—C41—H41C	109.5
C10—C11—H11	118.5	H41A—C41—H41C	109.5
C12—C11—H11	118.5	H41B—C41—H41C	109.5
C11—C12—C13	114.2 (4)	N3—C42—H42A	109.5
C11—C12—N1	110.3 (3)	N3—C42—H42B	109.5
C13—C12—N1	107.3 (3)	H42A—C42—H42B	109.5
C11—C12—H12	108.3	N3—C42—H42C	109.5
C13—C12—H12	108.3	H42A—C42—H42C	109.5
N1—C12—H12	108.3	H42B—C42—H42C	109.5
C12—C13—H13A	109.5	C44—C43—H43A	109.5
C12—C13—H13B	109.5	C44—C43—H43B	109.5
H13A—C13—H13B	109.5	H43A—C43—H43B	109.5
C12—C13—H13C	109.5	C44—C43—H43C	109.5
H13A—C13—H13C	109.5	H43A—C43—H43C	109.5
H13B—C13—H13C	109.5	H43B—C43—H43C	109.5
N1—C14—H14A	109.5	C45—C44—C43	122.0 (4)
N1—C14—H14B	109.5	C45—C44—C49	122.3 (4)
H14A—C14—H14B	109.5	C43—C44—C49	115.6 (3)
N1—C14—H14C	109.5	C44—C45—C46	123.0 (4)
H14A—C14—H14C	109.5	C44—C45—H45	118.5
H14B—C14—H14C	109.5	C46—C45—H45	118.5
C16—C15—H15A	109.5	O4—C46—C45	121.5 (4)
C16—C15—H15B	109.5	O4—C46—C47	121.4 (4)
H15A—C15—H15B	109.5	C45—C46—C47	117.0 (4)
C16—C15—H15C	109.5	C46—C47—C48	114.5 (3)
H15A—C15—H15C	109.5	C46—C47—H47A	108.6
H15B—C15—H15C	109.5	C48—C47—H47A	108.6
C17—C16—C15	123.4 (4)	C46—C47—H47B	108.6
C17—C16—C21	121.0 (4)	C48—C47—H47B	108.6
C15—C16—C21	115.6 (4)	H47A—C47—H47B	107.6
C16—C17—C18	123.4 (4)	C51—C48—C50	108.8 (3)
C16—C17—H17	118.3	C51—C48—C47	109.3 (3)
C18—C17—H17	118.3	C50—C48—C47	110.3 (3)
O2—C18—C17	121.8 (5)	C51—C48—C49	110.9 (3)
O2—C18—C19	120.8 (4)	C50—C48—C49	108.6 (3)
C17—C18—C19	117.4 (4)	C47—C48—C49	108.9 (3)
C18—C19—C20	113.7 (4)	C44—C49—C52	108.3 (3)
C18—C19—H19A	108.8	C44—C49—C48	111.3 (3)
C20—C19—H19A	108.8	C52—C49—C48	112.6 (3)
C18—C19—H19B	108.8	C44—C49—H49	108.2
C20—C19—H19B	108.8	C52—C49—H49	108.2
H19A—C19—H19B	107.7	C48—C49—H49	108.2
C23—C20—C22	109.7 (3)	C48—C50—H50A	109.5
C23—C20—C21	110.8 (3)	C48—C50—H50B	109.5
C22—C20—C21	109.2 (3)	H50A—C50—H50B	109.5
C23—C20—C19	109.0 (3)	C48—C50—H50C	109.5
C22—C20—C19	109.4 (3)	H50A—C50—H50C	109.5
C21—C20—C19	108.7 (3)	H50B—C50—H50C	109.5
C24—C21—C16	107.7 (3)	C48—C51—H51A	109.5
C24—C21—C20	112.7 (3)	C48—C51—H51B	109.5
C16—C21—C20	112.4 (3)	H51A—C51—H51B	109.5
C24—C21—H21	108.0	C48—C51—H51C	109.5
C16—C21—H21	108.0	H51A—C51—H51C	109.5
C20—C21—H21	108.0	H51B—C51—H51C	109.5
C20—C22—H22A	109.5	C53—C52—C49	126.0 (4)
C20—C22—H22B	109.5	C53—C52—H52	117.0
H22A—C22—H22B	109.5	C49—C52—H52	117.0
C20—C22—H22C	109.5	C52—C53—C54	121.6 (3)
H22A—C22—H22C	109.5	C52—C53—H53	119.2
H22B—C22—H22C	109.5	C54—C53—H53	119.2
C20—C23—H23A	109.5	C53—C54—N4	109.5 (3)
C20—C23—H23B	109.5	C53—C54—C55	114.0 (3)
H23A—C23—H23B	109.5	N4—C54—C55	107.8 (3)
C20—C23—H23C	109.5	C53—C54—H54	108.5
H23A—C23—H23C	109.5	N4—C54—H54	108.5
H23B—C23—H23C	109.5	C55—C54—H54	108.5
C25—C24—C21	126.6 (3)	C54—C55—H55A	109.5
C25—C24—H24	116.7	C54—C55—H55B	109.5
C21—C24—H24	116.7	H55A—C55—H55B	109.5
C24—C25—C26	122.3 (3)	C54—C55—H55C	109.5
C24—C25—H25	118.9	H55A—C55—H55C	109.5
C26—C25—H25	118.9	H55B—C55—H55C	109.5
C25—C26—N2	110.7 (3)	N4—C56—H56A	109.5
C25—C26—C27	113.4 (3)	N4—C56—H56B	109.5
N2—C26—C27	107.7 (3)	H56A—C56—H56B	109.5
C25—C26—H26	108.3	N4—C56—H56C	109.5
N2—C26—H26	108.3	H56A—C56—H56C	109.5
C27—C26—H26	108.3	H56B—C56—H56C	109.5
			
C1—C2—C3—C4	−179.1 (4)	C29—C30—C31—C32	177.6 (4)
C7—C2—C3—C4	−2.3 (6)	C35—C30—C31—C32	−2.3 (7)
C2—C3—C4—O1	178.2 (4)	C30—C31—C32—O3	−178.4 (4)
C2—C3—C4—C5	0.8 (6)	C30—C31—C32—C33	2.7 (7)
O1—C4—C5—C6	156.5 (4)	O3—C32—C33—C34	152.3 (4)
C3—C4—C5—C6	−26.1 (5)	C31—C32—C33—C34	−28.8 (5)
C4—C5—C6—C9	171.0 (3)	C32—C33—C34—C37	173.5 (3)
C4—C5—C6—C8	−69.2 (4)	C32—C33—C34—C36	−67.5 (4)
C4—C5—C6—C7	49.9 (4)	C32—C33—C34—C35	51.6 (4)
C3—C2—C7—C10	−95.1 (4)	C31—C30—C35—C38	−96.7 (5)
C1—C2—C7—C10	81.9 (4)	C29—C30—C35—C38	83.4 (4)
C3—C2—C7—C6	28.2 (5)	C31—C30—C35—C34	27.1 (5)
C1—C2—C7—C6	−154.8 (3)	C29—C30—C35—C34	−152.9 (4)
C9—C6—C7—C10	−50.5 (4)	C33—C34—C35—C38	72.2 (4)
C8—C6—C7—C10	−170.4 (3)	C37—C34—C35—C38	−48.6 (4)
C5—C6—C7—C10	70.2 (4)	C36—C34—C35—C38	−168.5 (3)
C9—C6—C7—C2	−170.7 (3)	C33—C34—C35—C30	−50.1 (4)
C8—C6—C7—C2	69.4 (4)	C37—C34—C35—C30	−170.8 (3)
C5—C6—C7—C2	−50.0 (4)	C36—C34—C35—C30	69.2 (4)
C2—C7—C10—C11	−103.9 (5)	C30—C35—C38—C39	−114.5 (4)
C6—C7—C10—C11	132.9 (4)	C34—C35—C38—C39	121.2 (4)
C7—C10—C11—C12	177.2 (4)	C35—C38—C39—C40	−178.5 (4)
C10—C11—C12—C13	−124.1 (4)	C38—C39—C40—C41	−124.9 (4)
C10—C11—C12—N1	115.0 (4)	C38—C39—C40—N3	114.2 (4)
C14—N1—C12—C11	−52.5 (5)	C42—N3—C40—C39	−55.9 (4)
C14—N1—C12—C13	−177.5 (3)	C42—N3—C40—C41	179.0 (3)
C15—C16—C17—C18	179.4 (4)	C43—C44—C45—C46	−179.6 (4)
C21—C16—C17—C18	−2.3 (6)	C49—C44—C45—C46	−2.9 (6)
C16—C17—C18—O2	−177.1 (4)	C44—C45—C46—O4	178.9 (4)
C16—C17—C18—C19	3.5 (6)	C44—C45—C46—C47	−0.4 (6)
O2—C18—C19—C20	150.8 (4)	O4—C46—C47—C48	156.0 (4)
C17—C18—C19—C20	−29.8 (5)	C45—C46—C47—C48	−24.6 (5)
C18—C19—C20—C23	173.3 (3)	C46—C47—C48—C51	171.2 (3)
C18—C19—C20—C22	−66.7 (4)	C46—C47—C48—C50	−69.1 (4)
C18—C19—C20—C21	52.5 (4)	C46—C47—C48—C49	50.0 (4)
C17—C16—C21—C24	−97.4 (5)	C45—C44—C49—C52	−94.0 (5)
C15—C16—C21—C24	80.9 (4)	C43—C44—C49—C52	82.8 (4)
C17—C16—C21—C20	27.3 (5)	C45—C44—C49—C48	30.3 (5)
C15—C16—C21—C20	−154.4 (3)	C43—C44—C49—C48	−152.9 (3)
C23—C20—C21—C24	−48.3 (4)	C51—C48—C49—C44	−171.9 (3)
C22—C20—C21—C24	−169.3 (3)	C50—C48—C49—C44	68.6 (4)
C19—C20—C21—C24	71.4 (4)	C47—C48—C49—C44	−51.6 (4)
C23—C20—C21—C16	−170.2 (3)	C51—C48—C49—C52	−50.1 (4)
C22—C20—C21—C16	68.8 (4)	C50—C48—C49—C52	−169.6 (3)
C19—C20—C21—C16	−50.5 (4)	C47—C48—C49—C52	70.2 (4)
C16—C21—C24—C25	−114.8 (4)	C44—C49—C52—C53	−106.0 (5)
C20—C21—C24—C25	120.7 (4)	C48—C49—C52—C53	130.5 (4)
C21—C24—C25—C26	−179.5 (4)	C49—C52—C53—C54	−178.5 (4)
C24—C25—C26—N2	115.7 (4)	C52—C53—C54—N4	114.6 (4)
C24—C25—C26—C27	−123.1 (4)	C52—C53—C54—C55	−124.7 (4)
C28—N2—C26—C25	−57.2 (4)	C56—N4—C54—C53	−53.5 (4)
C28—N2—C26—C27	178.3 (3)	C56—N4—C54—C55	−178.0 (3)

### Hydrogen-bond geometry (Å, °)

**Table:**

*D*—H···*A*	*D*—H	H···*A*	*D*···*A*	*D*—H···*A*
N1—H1A···Cl1^i^	0.93 (2)	2.19 (2)	3.109 (3)	173 (3)
N1—H1B···Cl4^i^	0.93 (2)	2.17 (2)	3.069 (4)	163 (3)
N2—H2A···Cl2	0.94 (2)	2.17 (2)	3.105 (3)	171 (3)
N2—H2B···Cl1	0.91 (2)	2.19 (2)	3.089 (4)	175 (4)
N3—H3A···Cl3^ii^	0.93 (2)	2.18 (2)	3.108 (3)	175 (3)
N3—H3B···Cl2^i^	0.92 (2)	2.18 (2)	3.088 (4)	171 (3)
N4—H4A···Cl4^iii^	0.85 (3)	2.28 (4)	3.117 (4)	169 (4)
N4—H4B···Cl3^iii^	0.96 (4)	2.13 (4)	3.083 (4)	172 (4)

Symmetry codes: (i) *x*, *y*, *z*−1; (ii) *x*+1, *y*, *z*−1; (iii) *x*+1, *y*, *z*.
